# Genomic and clinical characterization of *Klebsiella pneumoniae* carrying the *pks* island

**DOI:** 10.3389/fmicb.2023.1189120

**Published:** 2023-09-21

**Authors:** Zhiqian Wang, Yanjun Liu, Peilin Liu, Zijuan Jian, Qun Yan, Bin Tang, Awen Yang, Wenen Liu

**Affiliations:** ^1^Department of Clinical Laboratory, Xiangya Hospital, Central South University, Changsha, Hunan, China; ^2^National Clinical Research Center for Geriatric Disorders, Xiangya Hospital, Changsha, Hunan, China

**Keywords:** *Klebsiella pneumoniae*, *pks* island, phylogeny, mobile genetic elements, clinical characteristics

## Abstract

**Background:**

The *pks* island and its production of the bacterial secondary metabolite genotoxin, colibactin, have attracted increasing attention. However, genomic articles focusing on *pks* islands in *Klebsiella pneumoniae*, as well as comparative genomic studies of mobile genetic elements, such as prophages, plasmids, and insertion sequences, are lacking. In this study, a large-scale analysis was conducted to understand the prevalence and evolution of *pks* islands, differences in mobile genetic elements between *pks*-negative and *pks*-positive *K. pneumoniae*, and clinical characteristics of infection caused by *pks*-positive *K. pneumoniae.*

**Methods:**

The genomes of 2,709 *K. pneumoniae* were downloaded from public databases, among which, 1,422 were from NCBI and 1,287 were from the China National GeneBank DataBase (CNGBdb). Screening for virulence and resistance genes, phylogenetic tree construction, and pan-genome analysis were performed. Differences in mobile genetic elements between *pks*-positive and *pks*-negative strains were compared. The clinical characteristics of 157 *pks*-positive and 157 *pks*-negative *K. pneumoniae* infected patients were investigated.

**Results:**

Of 2,709 *K. pneumoniae* genomes, 245 *pks*-positive genomes were screened. The four siderophores, type VI secretion system, and nutritional factor genes were present in at least 77.9% (191/245), 66.9% (164/245), and 63.3% (155/245) of *pks*-positive strains, respectively. The number and fragment length of prophage were lower in *pks*-positive strains than in *pks*-negative strains (*p* < 0.05). The prevalence of the IS6 family was higher in *pks*-negative strains than in *pks*-positive strains, and the prevalence of multiple plasmid replicon types differed between the *pks*-positive and *pks*-negative strains (*p* < 0.05). The detection rate of *pks*-positive *K. pneumoniae* in abscess samples was higher than that of *pks*-negative *K. pneumoniae* (*p* < 0.05).

**Conclusion:**

The *pks*-positive strains had abundant virulence genes. There were differences in the distribution of mobile genetic elements between *pks*-positive and *pks*-negative isolates. Further analysis of the evolutionary pattern of *pks* island and epidemiological surveillance in different populations are needed.

## 1. Introduction

*Klebsiella pneumoniae* is a common opportunistic pathogen in hospitals, that can cause a variety of serious infections (Siu et al., 2012). In recent years, *K. pneumoniae* has become a major threat to global public health. Although many factors, including host immunity (Bengoechea and Sa Pessoa, 2019), contribute to the pathogenicity of *K. pneumoniae* in humans, virulence factors undoubtedly play a critical role in the pathogenesis process. *K. pneumoniae* includes two different phenotypes: classical *K. pneumoniae* (cKp) and hypervirulent *K. pneumoniae* (hvKp). Hypervirulent *K. pneumoniae* (hvKp) can cause severe infection in immunocompetent individuals with high pathogenicity and mortality (Russo and Marr, 2019). Factors contributing to the high virulence of the hvKp include capsule, siderophores, lipopolysaccharide and fimbriae (Zhu et al., 2021). Recently, *pks* islands, which are located on the same integrative and conjugative element as the yersiniabactin gene cluster, have been considered as a new virulence factor and have attracted more and more attention.

The *pks* island, also known as the *clb* gene island, consists of 19 genes (*clbA-clbS*). It was first identified in *Escherichia coli* IHE3034 (Nougayrède et al., 2006) and was later found in other enterobacteria, including *K. pneumoniae*. Colibactin is a genotoxic bacterial secondary metabolite produced by the *pks* island, and it can interfere with the cell cycle in eukaryotic cells and cause cell damage; consequently, the *pks* locus-mediated colibactin production can enhance the virulence and pathogenicity of bacteria. *E. coli* carrying the *pks* island may be involved in the development and progression of colorectal cancer and exacerbate lymphocytopenia associated with sepsis (Marcq et al., 2014; Prorok-Hamon et al., 2014; Pleguezuelos-Manzano et al., 2020). Hypervirulent *K. pneumoniae* carrying the *pks* gene island induced meningitis successfully in mice, whereas deletion of *clbA* gene and abolition of colibactin synthesis significantly reduced the ability of the bacterium to cause meningitis in mice (Lu et al., 2017).

Horizontal gene transfer is an essential aspect in bacterial evolution and is often associated with mobile genetic elements (MGEs) (Koonin et al., 2001; Soucy et al., 2015; Haudiquet et al., 2022). Previous studies have reported a high degree of consistency in *pks* islands between different bacterial species, and *pks* islands are characterized for being acquired by horizontal gene transfer (Putze et al., 2009). Many genomic studies focusing on *E. coli* have shown the horizontal propagation and evolution pattern of *pks* islands in *E. coli* and revealed the different acquisitions of *pks* islands (Auvray et al., 2021; Suresh et al., 2021; Wami et al., 2021). However, there are limited information focusing on *pks*-positive *K. pneumoniae*. Genomic analyses of *K. pneumoniae* have shown that *pks* islands and *ybt* genes can be horizontally transferred between *K. pneumoniae* by integrative and conjugative elements (ICEs) (Lam et al., 2018). This study aimed to comprehensively understand the prevalence and evolution of *pks* island in *K. pneumoniae*, compare the differences in mobile genetic elements between *pks*-positive and *pks*-negative *K. pneumoniae*, and investigate the clinical characteristics of *pks*-positive *K. pneumoniae* infection. This study will broaden our conceptual understanding of the disease-causing *K. pneumoniae pks*-positive strains and could facilitate the improvement of diagnosis and characterization of associated infections.

## 2. Materials and methods

### 2.1. *Klebsiella pneumoniae* genome sequences utilized in this study

A total of 2,709 *K. pneumoniae* genome sequences (1,287 complete genomes and 1,422 draft genomes) from two public databases were screened in this study. Among these sequences, 1,287 clinical strain high-quality genome sequences from a large tertiary hospital from January 2016 to July 2018 were downloaded from the China National GenBank DataBase (Chen et al., 2020).^1^ The remaining 1,422 *K. pneumoniae* genome sequences were downloaded from the NCBI database (accessed on October 13, 2022). The accession numbers of all the genes are shown in Supplementary Table S1.

### 2.2. Identification of *pks*-positive genomes

The *pks* sequence of *K. pneumoniae* strain 1,084 (CP003785.1) was used as the reference sequence. The *pks*-positive genomes were identified by comparing the reference sequence with the 2,709 *K. pneumoniae* genome using BLAST+ 2.9.0. The identity and query coverage thresholds for BLAST+ 2.9.0 were set at 90%. The identified *pks*-positive genomes were further confirmed using Kleborate version 2.2.0 (Lam et al., 2021).

### 2.3. Multi-locus sequence typing and serotype analysis

The sequence types of *pks*-positive *K. pneumoniae* were determined using MLST version 2.22.0,^2^ which can scan genome sequences against the traditional PubMLST typing schemes.^3^ Kleborate version 2.2.0 (Lam et al., 2021) was used to analyze the allelic differences of *pks* island and yersiniabactin determinant in each genome to determine colibactin and yersiniabactin determinant sequence types (CbST and YbST). In addition, the serotypes of *pks*-positive strains were identified using the kleborate version 2.2.0 (Lam et al., 2021).

### 2.4. Analysis of virulence genes and antibiotic resistance genes

All *pks-*positive and *pks*-negative *K. pneumoniae* strains were used for further analyses. Virulence and resistance genes were identified by comparison with the Virulence Factor Database (VFDB) and the Comprehensive Antibiotic Resistance Database (CARD) using abricate version 1.0.1^4^ with default settings (Chen et al., 2005; McArthur et al., 2013). Databases used to compare virulence and resistance genes were updated to the latest version before the analysis.

### 2.5. Pangenome analysis of *Klebsiella pneumoniae* strains

All *K. pneumoniae* strains were annotated using Prokka version 1.14 (Seemann, 2014). The annotation files of *pks*-positive strains obtained in the previous step were further analyzed using Roary version 3.13.0 (Page et al., 2015) with default settings to obtain the core genes of the positive strains. Genes present in at least 99% of the strains were identified as core genes. Clusters of Orthologous Genes (COG) classification was obtained by uploading the core genes to the eggNOG (Evolutionary Genealogy of Genes: Non-supervised Orthologous Groups) public database (Huerta-Cepas et al., 2016).

### 2.6. Phylogenetic analysis

The *pks*-positive strains were aligned to the reference genome *K. pneumoniae* 1,084 (CP003785.1) using Snippy version 4.6.0,^5^ and then the core gene SNP sites were extracted. After removing the recombinant regions using Gubbins version 2.4.1 (Croucher et al., 2015), phylogenetic trees using the maximum likelihood method were constructed using FastTree version 2.1.11 and uploaded to the iTOL website for annotation (Price et al., 2009; Letunic and Bork, 2021). A phylogenetic tree based on the *pks* island was constructed by aligning the genomic sequences of *pks*-positive strains to the *pks* island sequence of the reference strain. The phylogenetic tree based on core genes of all *pks*-positive strains and that based on the *pks* island were linked using the R package Phytools to construct the co-phylogenetic tree (Revell, 2012).

### 2.7. Identification of mobile genetic elements

The genome sequences of the strains were uploaded to the ICEFinder and ISFinder websites to predict the number and types of integrative and conjugative elements (ICEs) and insertion sequences (ISs) of *K. pneumoniae* strains (Siguier et al., 2006; Liu et al., 2019). The *e* value of the ISfinder was set to 1e-5. The gene sequences were submitted to the PHASTER website to determine the number and size of prophages (Arndt et al., 2016). The plasmid replicon type was determined using staramr version 0.8.0 (Bharat et al., 2022), which can scan the bacterial genome against the PlasmidFinder database (Carattoli et al., 2014). The percent identity threshold and percent length overlap for the PlasmidFinder results were set to 98% and 60%, respectively.

### 2.8. Collection of clinical information

Clinical data of 157 *pks*-positive and 157 *pks*-negative *K. pneumoniae* patients (random sampling) from a large tertiary hospital in China were collected by consulting the electronic medical records. Specifically, stratified random sampling was used to enroll patients in this study. Clinical data from all 157 patients with *pks*-positive *K. pneumoniae* infection in this hospital were included in the study. The number of enrolled patients with *pks*-negative *K. pneumoniae* infection was determined at a 1:1 ratio. Considering that differences in the prevalence of *K. pneumoniae* infection may occur in different years and in different quarters, we stratified the patients with *pks*-negative infection in this hospital by year and month. Then, according to the year and month distribution of *pks*-positive *K. pneumoniae* infection patients, the number of *pks*-negative *K. pneumoniae* infection enrolled patients in each year and different months of each year was determined. Within each stratum, random sampling was performed according to computer-generated random numbers. The clinical information collected included sex, age, underlying conditions, sample source, and ward distribution.

### 2.9. Statistical analysis

Categorical variables were analyzed using the chi-square test or Fisher’s exact test. Continuous variables were analyzed using *t* test or Mann–Whitney U test. Statistical analyses of all data were performed on GraphPad Prism 9 and SPSS version 26.0. *p* < 0.05 was considered statistically significant.

### 2.10. Ethics statement

This study was approved by the Central South University Ethics Committee (Changsha, Hunan Province, People’s Republic of China) with ID 202212294.

## 3. Results

### 3.1. Prevalence and distribution of *pks*-positive *Klebsiella pneumoniae*

**Figure 1:**
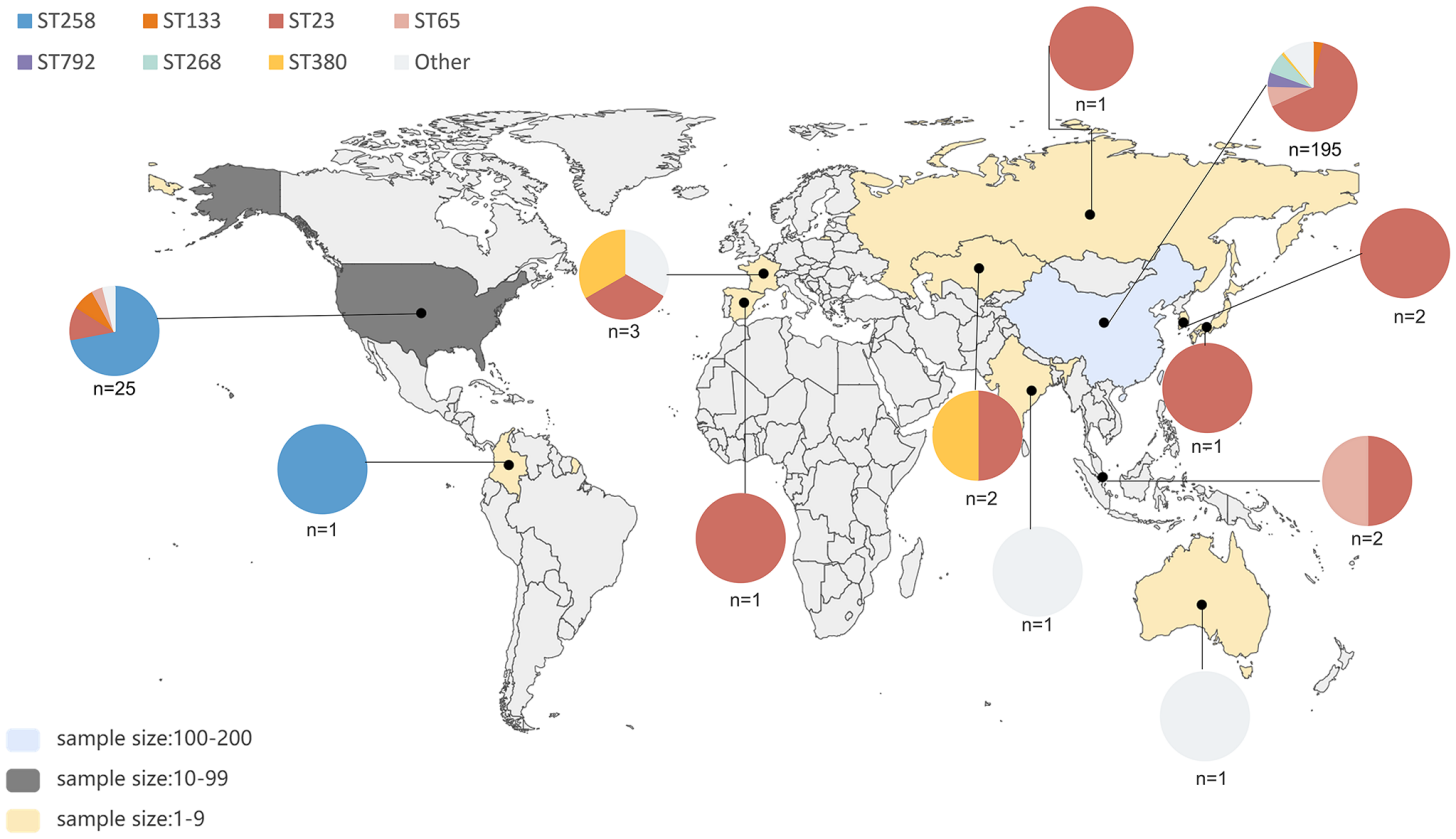
Geographic distribution and sequence type distribution of 235 *pk*s-positive strains. A total of 245 *pks*-positive strains were screened, of which 10 had unknown background information. Sequence types and their proportions of *pks*-positive strains in each region are indicated by different colors in the pie chart. The number of *pks*-positive strains in different regions of this study is indicated by different colors, as shown in the bottom left corner.

A total of 245 *pks*-positive *K. pneumoniae* genomes were identified from 2,709 genomes; of them, 157 were from the China National GeneBank Database (CNGBdb) (Chen et al., 2020) and 88 were from the public database NCBI. The 245 *pks*-positive *K. pneumoniae* included 79.6% (195/245) from China, 10.2% (25/245) from the United States, 6.1% (15/245) from other countries, and 4.1% (10/245) with unknown background information (Figure 1). Multi-locus sequence typing showed that 58.0% (142/245) of *pks*-positive *K. pneumoniae* were ST23 and 8.2% (20/245) were ST258. ST65, ST268, and ST792 accounted for 6.9% (17/245), 6.9% (17/245), and 4.5% (11/245) of the 245 *pks*-positive strains, respectively. The main serotype was K1, accounting for 63.3% (155/245) of cases. K2, K20, and K107 accounted for 10.6% (26/245), 6.9% (17/245), and 7.3% (18/245) of the 245 pk-positive strains, respectively (Table 1).

**Table 1:** Sequence type and serotype distribution of 245 *pks*-positive *K. pneumoniae*.

Characteristics	*n* (%)
Sequence type	
ST23	142 (58.0)
ST258	20 (8.2)
ST65	17 (6.9)
ST268	17 (6.9)
ST792	11 (4.5)
ST133	10 (4.1)
ST380	4 (1.6)
Other	24 (9.8)
K serotype	
K1	155 (63.3)
K2	26 (10.6)
K20	17 (6.9)
K107	18 (7.3)
Other	29 (11.8)

### 3.2. Distribution of virulence and antibiotic resistance genes in *pks*-positive strains

**Figure 2:**
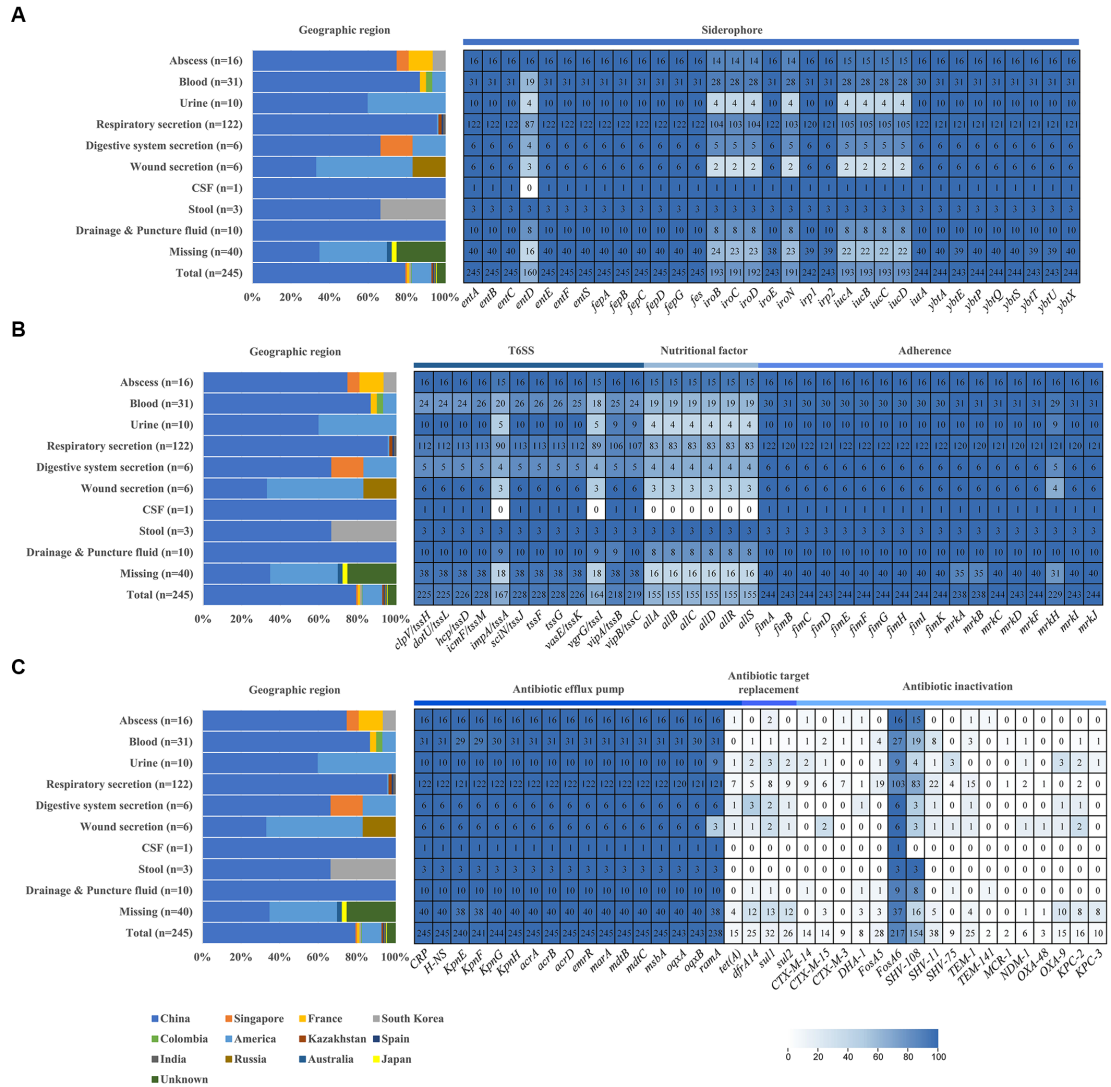
Distribution of geographical, virulence genes, and antibiotic resistance genes in *pks*-positive *Klebsiella pneumoniae* from different sources. **(A)** Siderophore associated virulence gene. **(B)** T6SS, nutritional factor and adherence. **(C)** Antibiotic resistance genes. Graphs and heatmaps show the proportion of strains, and the numbers in the heatmap boxes represent the number of strains harboring the virulence or antibiotic resistance gene.

To determine the prevalence of virulence and resistance genes, we screened the genomes of 245 *pks*-positive *K. pneumoniae* (Figure 2). Virulence genes, including siderophores, type VI secretory system, type 1 and type 3 fimbriae, and nutritional factors, were investigated in this study. Yersiniabactin siderophore system genes (*ybtAEPQSTUX*, *irp1*, *irp2*) and enterobactin synthase genes (*entABCEFS, fepABCDG*) were found in at least 98.7% (242/245) of the 245 *pks*-positive strains. Aerobactin siderophore synthesis system genes (*iucABCD*) and salmochelin genes (*iroBCDEN*) were present in at least 77.9% (191/245) of the 245 *pks*-positive strains (Figure 2A). Notably, *pks*-positive strains showed a high prevalence of type VI secretion systems. Type VI secretion system-associated genes such as membrane complex-associated genes (*tssL, tssM*), substrate complex-associated genes (*tssF, tssG, tssK*), and tail complex-associated genes (*tssA, tssB, tssC, tssD*) were present in at least 66.9% (164/245) of the 245 *pks*-positive strains. The type 3 fimbriae gene (*mrkABCDFHIJ*) and type 1 fimbriae gene (*fimABCEFGHIK*) were present in at least 93.5% (229/245) of the 245 *pks*-positive strains. Nutritional factor genes (*allABCDRS*) were present in 63.3% (155/245) of the 245 *pks*-positive strains (Figure 2B).

Compared with *pks*-positive *K. pneumoniae*, the prevalence of these virulence genes in *pks*-negative *K. pneumoniae* was lower. Among 2,464 *pks*-negative isolates, yersiniabactin siderophore system genes (*ybtAEPQSTUX, irp1, irp2*), aerobactin siderophore synthesis system genes (*iucABCD*) and salmochelin genes (*iroBCDEN*) were detected in no more than 51.2% (1,261/2464), 23.0% (566/2464) and 10.5% (259/2464) of genomes, respectively. Nutritional factor genes (*allABCDRS*) and type VI secretory system-associated genes (*impA/tssA, vgrG/tssI*) were found in 2.2% (55/2464) and 30.3% (746/2464) of the 2,464 *pks*-negative strains, respectively (Supplementary Figure S1).

Antibiotic resistance genes associated with efflux pumps, antibiotic target replacement, and antibiotic inactivation were investigated (Figure 2C). Efflux pump genes, including *CRP* (global regulator), *acr* (aminoglycoside efflux pump), *mar* (multiple antibiotic resistance family), *mdt* (multi-drug efflux system), *oqxAB* (mediating quinolone resistance) and *KpnEFGH*, were present in at least 97.1% (238/245) of 245 *pks*-positive strains. Antibiotic target replacement genes (*dfrA14, sul1, and sul2*) were present in no more than 13.1% (32/245) of the 245 *pks*-positive strains. OXA-β-lactamase, NDM, KPC, and CTX-M-β-lactamase, which belong to antibiotic inactivation genes, were present in 6.5% (16/245) of the 245 *pks*-positive strains. More detailed results of the antibiotic-resistance genes are shown in Figure 2C.

### 3.3. Phylogenetic analysis and pangenome analysis

**Figure 3:**
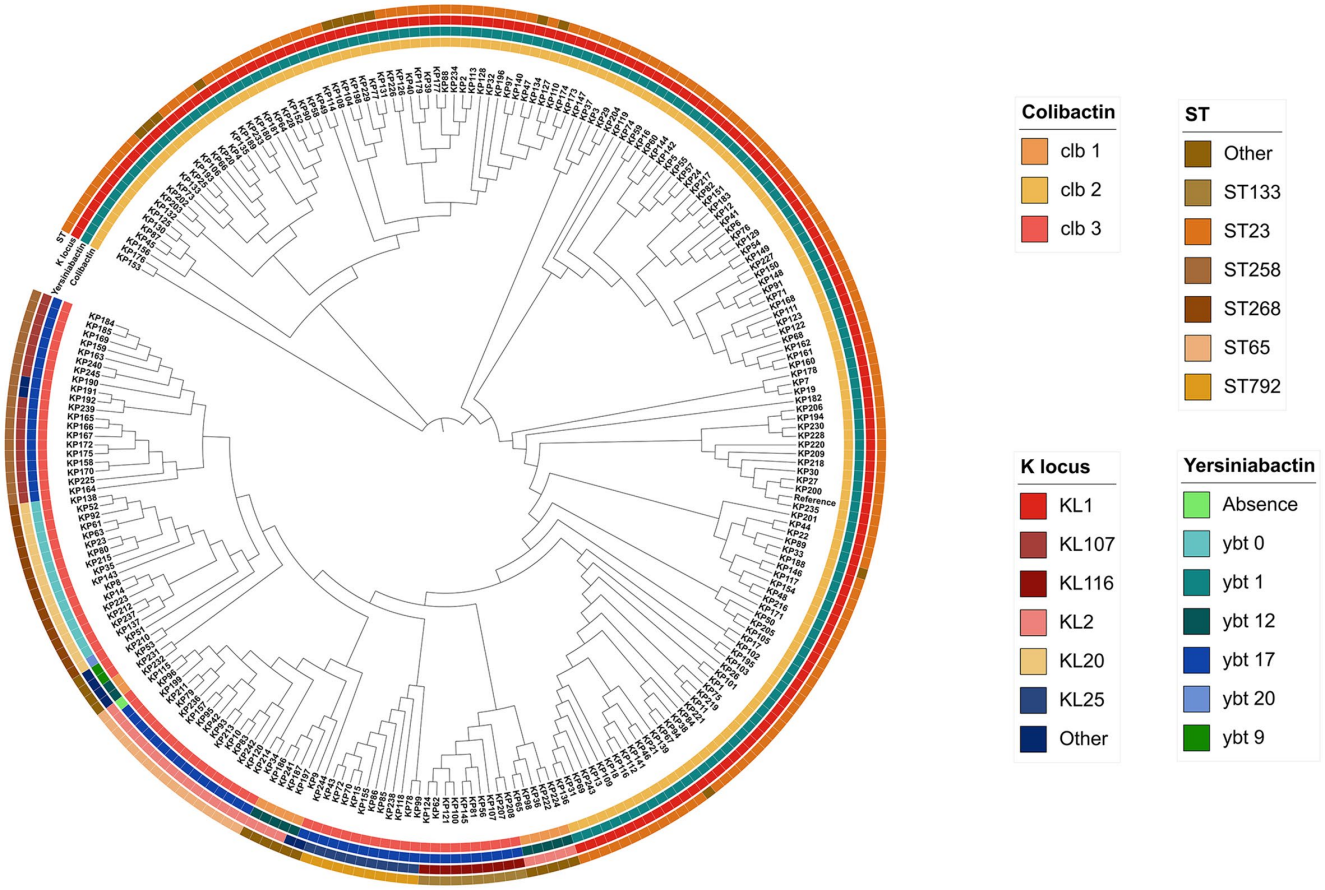
Phylogenetic tree based on SNP sites in core genes of 245 *pks*-positive strains. The outer circles from inside to outside indicate colibactin, yersiniabactin, serotype, and sequence type, respectively. Different colors in each circle represent different categories.

We constructed a maximum likelihood phylogenetic tree based on 245 *pks*-positive *K. pneumoniae* core genes (Figure 3). The 245 *pks*-positive *K. pneumoniae* strains have high genetic diversity and can be divided into different branches. Colibactin (*clb*) and yersiniabactin (*ybt*) determinants were strongly associated, and 99.6% (244/245) of the *pks*-positive strains carried both the *clb* and *ybt* genes (the first and second circles of the phylogenetic tree). In most cases, the distribution of the *ybt* locus and the *clb* locus is identical, with each *ybt* classification corresponding to a distinct *clb* classification.

We also identified 3,649 core genes (genes present in at least 99% of the strains were considered core genes) in 245 *pks*-positive *K. pneumoniae* using the Roary version 3.13.0 (Page et al., 2015). A total of 99.0% (3,614/3649) of the core genes were assigned to COG categories (some genes were assigned to more than one COG category; Supplementary Figure S2). As shown in Supplementary Figure S2, the core genes belonging to the S (Function unknown) category were the most abundant (*n* = 728) and the K (transcription) category was the second most abundant COG assigned category (*n* = 370). Cell cycle control, cell division, chromosome partitioning (D), cell motility (N), and defense mechanisms (V), which are cellular processes and signals, are less represented in the core genes (*n* = 51, *n* = 38, *n* = 42).

### 3.4. Evolution of the *pks* locus in *Klebsiella pneumoniae*

**Figure 4:**
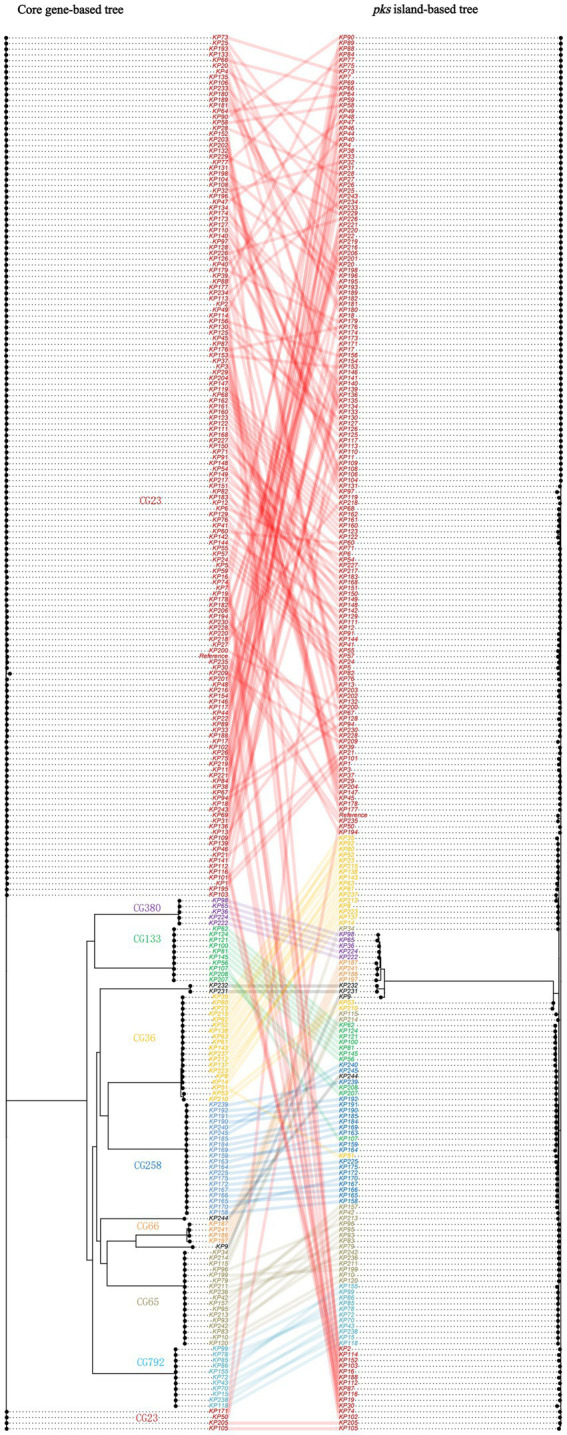
Co-phylogeny of *pks* sequences and *K. pneumoniae* strains. The strains belonging to the major clonal groups (CGs) are shown with colored names.

To further understand the scenario of strains acquiring *pks* sequences, we constructed a phylogenetic tree based on the *pks* sequence and connected it to a phylogenetic tree based on the core genes of *pks*-positive *K. pneumoniae* (Figure 4). It can be observed that the clustering patterns of strains of clonal group (CG) 380 and CG792 lineages are consistent in the two evolutionary trees. In comparison, the clustering patterns of strains of CG65 and CG23 lineages differ in the two evolutionary trees.

### 3.5. Differences between mobile genetic elements in *pks*-positive and *pks*-negative strains

**Figure 5:**
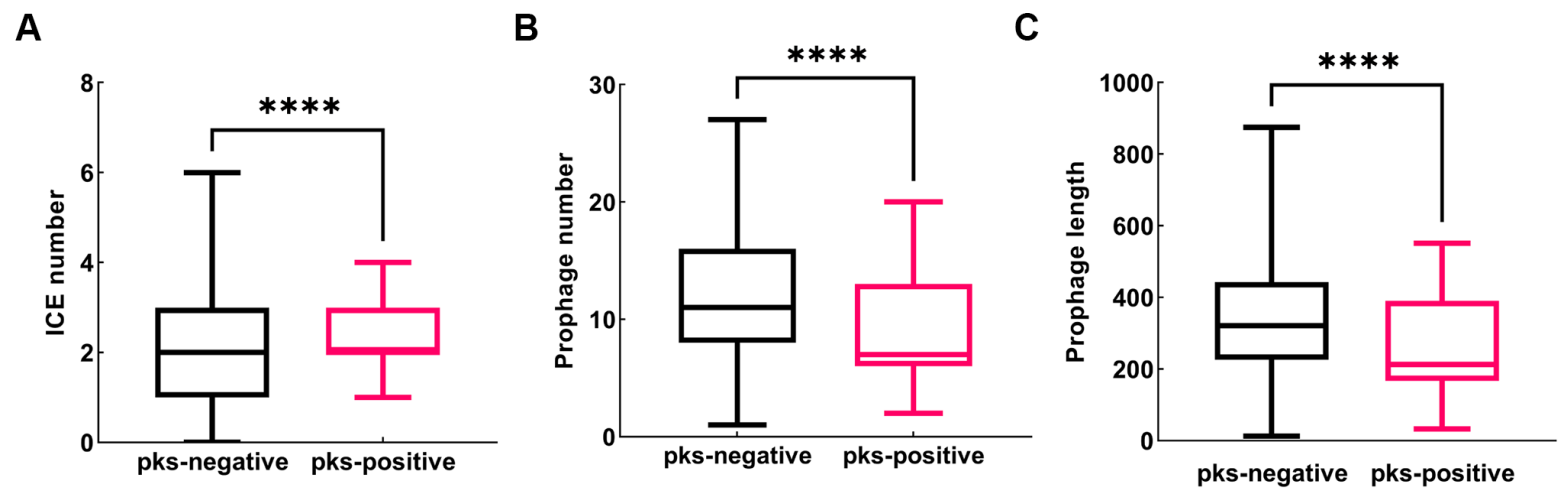
Distribution of predicted ICE and prophage in *pks*-positive and *pks*-negative *K. pneumoniae* genomes (complete genome). **(A)** The number of predicted ICE in the genomes of *pks*-positive and *pks*-negative strains. **(B)** The number of predicted prophage in the genomes of *pks*-positive and *pks*-negative strains. **(C)** The length of predicted prophage in the genomes of *pks*-positive and *pks*-negative strains.

**Figure 6:**
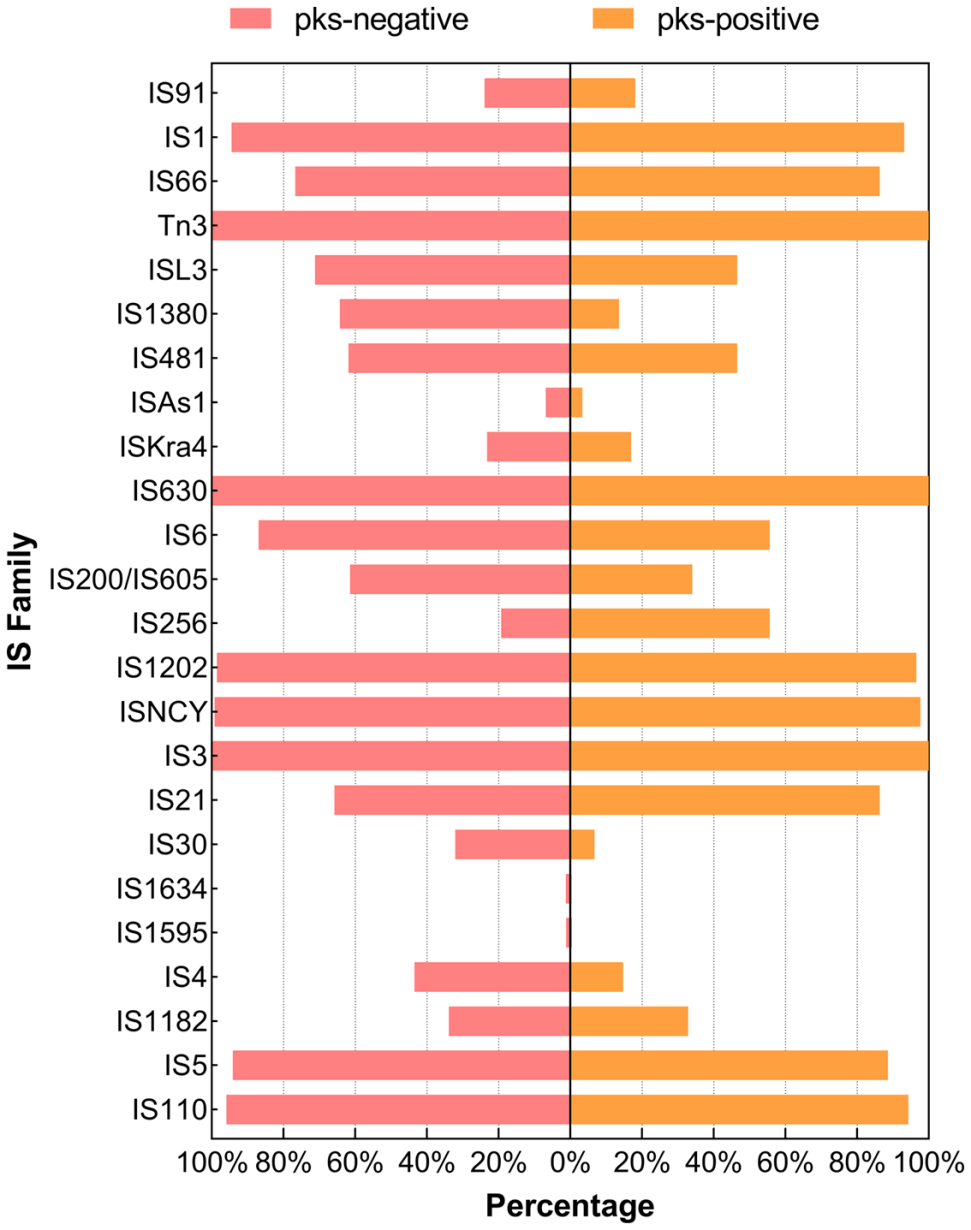
Distribution of the IS family between *pks*-positive and *pks*-negative *K. pneumoniae* (complete genome). The red color indicates that the distribution of this category in *pks*-positive and *pks*-negative strains is statistically significant.

**Figure 7:**
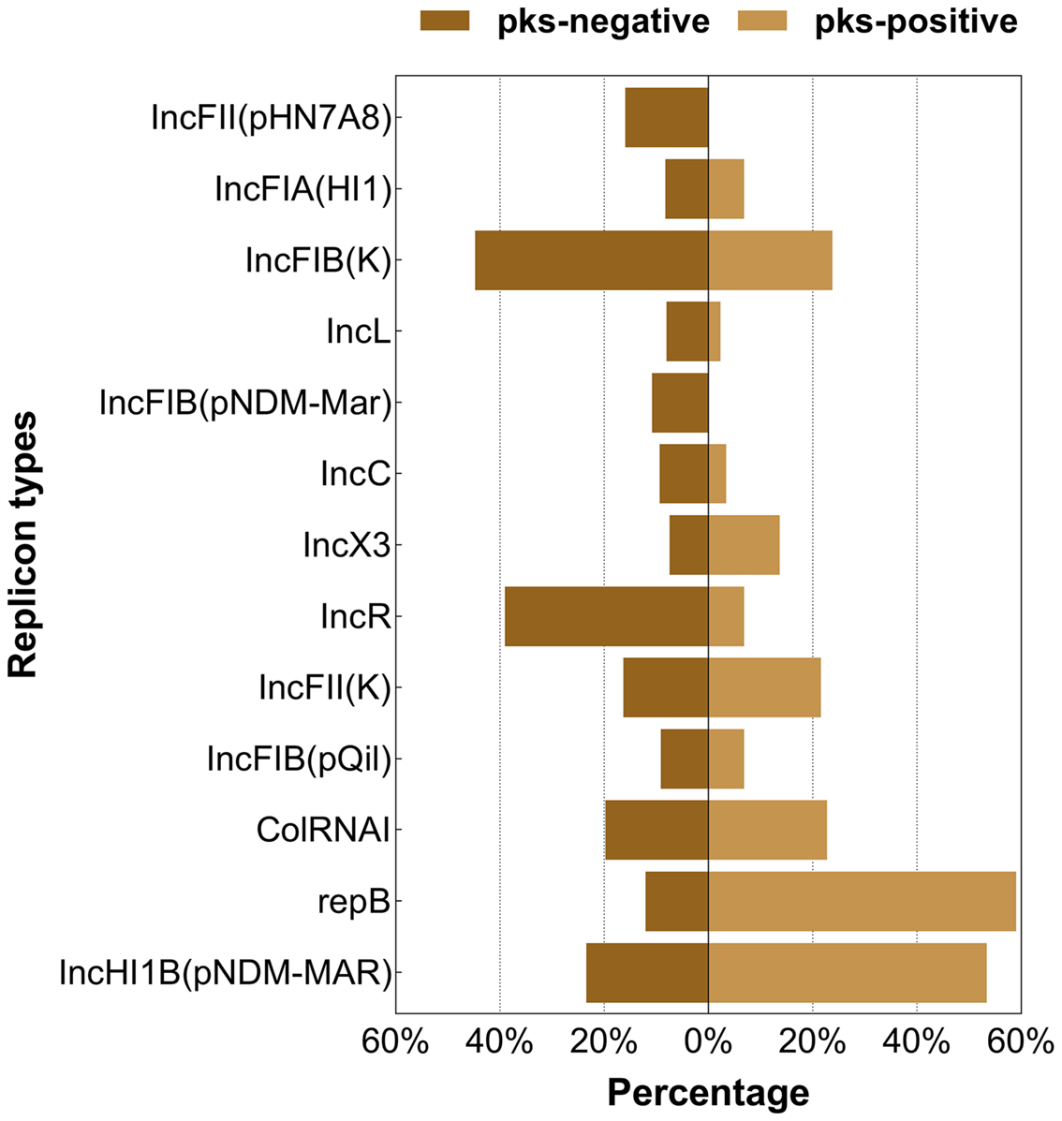
Distribution of the major plasmid replicon types between *pks*-positive and *pks*-negative *K. pneumoniae* (complete genome). The red color indicates that the distribution of this category in *pks*-positive and *pks*-negative strains is statistically significant.

To further understand the genetic background, we compared the distribution of mobile genetic elements, such as plasmids, insertion sequences (ISs), prophages, and integrative and conjugative elements (ICEs) in *pks*-positive and *pks*-negative *K. pneumoniae*. Considering the high accuracy of the complete genome, we focused our analysis on the mobile genetic elements of 88 *pks*-positive and 1,334 *pks*-negative *K. pneumoniae* complete genomes downloaded from NCBI (Figures 5–7). All 88 *pks*-positive strains carried at least one ICE, whereas 91.3% (1,218/1334) of *pks*-negative strains carried at least one ICE. However, the median number of ICEs carried by both *pks*-negative and *pks*-positive strains was 2 (Figure 5A). Prophages (intact, incomplete, and suspicious phages) were predicted in both *pks*-positive and *pks*-negative genomes. Notably, there were significant differences in the number and length of prophages between the *pks*-positive and *pks*-negative strains (Figures 5B,C). The median number of prophages in the *pks*-positive group was 7, while the median number of prophages in the *pks*-negative group was 11. Further, *pks*-negative *K. pneumoniae* appeared to carry longer prophage fragments than did *pks*-positive *K. pneumoniae* (median length 321.0 kb and 212.8 kb, respectively).

We predicted 24 insertion sequence families in *pks*-negative strains, but only 22 in *pks*-positive strains (Figure 6). IS*3*, IS*630*, Tn*3*, IS*1202*, and IS*NCY* were present in almost all 1,442 strains, while a variety of insertion sequences, including IS*6*, IS*L3*, IS*66*, and IS*1380*, were significantly different between *pks*-positive and *pks*-negative strains (*p* < 0.05). In addition, we found that the *pks*-negative strains had more plasmid replicon types than did the *pks*-positive strains, which may be due to the larger number of strains contained in the *pks*-negative group (Supplementary Table S2). The major plasmid replicon types and their proportions in both the groups are shown in Figure 7. These plasmid replicon types included incompatibility plasmids (Inc.) and mobilizable colicin plasmid (Col) groups. RepB (59.1%, 52/88) was the most abundant plasmid replicon type in the *pks*-positive strains, whereas the plasmid replicon type IncFIB(K) (44.8%, 597/1334) was the most abundant in the *pks*-negative strains. The distribution of several types of plasmid replicons, including IncR, IncFII(pHN7A8), IncFIB(pNDM-Mar), and IncX3, differed between *pks*-positive and *pks*-negative strains (*p* < 0.05). The differences in mobile genetic elements between *pks*-positive and *pks*-negative strains described above were similarly observed when the study was extended from complete genomes to include both complete and incomplete genomes (Supplementary Figure S3; Supplementary Tables S3, S4; Table 2).

**Table 2:** Distribution of plasmid replicon types in *pks*-positive and *pks*-negative *K. pneumoniae* genomes (complete and incomplete genomes are included).

Replicon types	*pks*-negative (*n* = 2,464)	*pks*-positive (*n* = 245)	*p*
IncFIB(K)	901 (36.6)	39 (15.9)	0.000*
IncHI1B(pNDM-MAR)	618 (25.1)	167 (68.2)	0.000*
repB	460 (18.7)	187 (76.3)	0.000*
IncFII(pHN7A8)	498 (20.2)	4 (1.6)	0.000*
IncR	951 (38.6)	25 (10.2)	0.000*
IncFIB(pNDM-Mar)	168 (6.8)	0 (0.0)	0.000*

*A *p* value < 0.05 was considered to be statistically significant. Only the plasmid replicon types that differed in the complete genome of *pks*-positive and *pks*-negative strains were analyzed statistically.

### 3.6. Clinical characteristics of *pks*-positive *Klebsiella pneumoniae* infection

We analyzed the clinical data of 157 *pks*-positive *K. pneumoniae* and 157 *pks*-negative *K. pneumoniae* infected patients to obtain a comprehensive picture of the risk factors for *pks*-positive *K. pneumoniae* infections (Tables 3, 4). The clinical characteristics associated with infection with *pks*-positive and *pks*-negative strains were different in several aspects. In infants younger than or equal to 1 year of age, the detection rate of *pks*-positive *K. pneumoniae* (1.9%, 3/157) was lower than that of *pks*-negative (12.1%, 19/157) *K. pneumoniae* (*p* < 0.05). Specimens from abscess sites were more likely to contain *pks*-positive strains (7.6%, 12/157) than *pks*-negative (1.9%, 3/157) strains (*p* < 0.05). Notably, the detection rate of *pks*-positive strains (1.9%, 3/157) was lower than that of *pks*-negative (11.5%, 18/157) strains in the neonatal ward (*p* < 0.05). This also corresponds to the lower incidence of infections caused by *pks*-positive *K. pneumoniae* in infants younger than or equal to 1 year of age, as described above. In addition, *pks*-positive strains (26.1%, 41/157) were more likely to be detected in patients from internal medicine than were *pks*-negative (14.6%, 23/157) strains (*p* < 0.05). Further, the statistical results suggested that patients with diabetes and cerebral infarction were more sensitive to *pks*-positive strains than *pks*-negative strains (*p* < 0.05).

**Table 3:** Demographic characteristics and underlying conditions of patients with *pks*-positive and *pks*-negative *K. pneumoniae* infection.

Characteristics	*pks*-positive (*n* = 157)	*pks*-negative (*n* = 157)	*p*
Male (*n*,%)	108 (68.8)	104 (66.2)	0.630
Age (*n*,%)			0.002*
<=1 y.o (22)	3 (1.9)	19 (12.1)	0.000*
1-64 y.o (214)	111 (70.7)	103 (65.6)	0.333
> = 65 y.o (78)	43 (27.3)	35 (22.3)	0.296
Underlying condition (*n*,%)			
Hypertension	46 (29.3)	33 (21.0)	0.091
Diabetes mellitus	40 (25.5)	20 (12.7)	0.004*
Biliary tract disease	11 (7.0)	17 (10.8)	0.235
Cancer	29 (18.5)	24 (15.3)	0.451
Autoimmune disease	7 (4.5)	3 (1.9)	0.199
Cerebral infarction	26 (16.6)	14 (8.9)	0.042*

*A *p* value < 0.05 was considered to be statistically significant.

**Table 4:** The specimen source and ward distribution of *pks*-positive and *pks*-negative isolates.

Characteristics	*pks*-positive (*n* = 157)	*pks*-negative (*n* = 157)	*p*
Specimen source (*n*,%)			
Respiratory secretion	105 (66.9)	94 (60.0)	0.198
Blood	18 (11.5)	23 (14.6)	0.402
Drainage & puncture fluid	10 (6.4)	12 (7.6)	0.658
Urine	6 (3.8)	10 (6.4)	0.305
Digestive system secretion	4 (2.5)	6 (3.8)	0.520
Abscess	12 (7.6)	3 (1.9)	0.017*
Wound secretion	1 (0.6)	7 (4.4)	0.067
CSF	1 (0.6)	2 (1.3)	1.000
Ward distribution (*n*,%)			
Pediatrics	2 (1.3)	5 (3.2)	0.448
Neonatal ward	3 (1.9)	18 (11.5)	0.001*
ICU	44 (28.0)	43 (27.4)	0.900
Medicine	41 (26.1)	23 (14.6)	0.012*
Integrated traditional Chinese & western medicine	5 (3.2)	6 (3.8)	0.759
Surgery	40 (25.5)	35 (22.3)	0.508
Oncology	4 (2.5)	3 (1.9)	1.000
Outpatient	9 (5.7)	10 (6.4)	0.813
Infectious diseases	3 (1.9)	3 (1.9)	1.000
Obstetrics & gynecology	3 (1.9)	7 (4.5)	0.199
Rehabilitation	3 (1.9)	4 (2.5)	1.000

*A *p* value < 0.05 was considered to be statistically significant.

## 4. Discussion

Here, a genomic analysis of *K. pneumoniae* genome sequences—obtained from public databases—was conducted and the clinical information of patients with *pks*-positive and *pks*-negative *K. pneumoniae* infections was investigated; consequently, the results provide new insights for a better understanding of the evolution and prevalence of the *pks* gene cluster in *K. pneumoniae.*

Studies have shown that K1 and ST23 are the main serotypes and sequence types of hypervirulent *K. pneumoniae* (hvKP) respectively (Struve et al., 2015; Shi et al., 2018). Our study found that K1 and ST23 were also the main serotypes and sequence types of *pks*-positive strains, which is consistent with results of previous studies (Chen et al., 2017; Lan et al., 2019; Shi et al., 2020). The sequence type of some of the *pks*-positive strains in our study was ST258. Notably, ST258 is one of the major sequence types of multi-drug resistant strains (Wyres et al., 2020). The presence of the *pks* island in the ST258 strains may reflect the unity of high virulence and resistance. In addition, 66.9% of *pks*-positive isolates contained type VI secretion system genes associated with hypervirulence. These suggest that the *pks* gene cluster and high virulence genes are closely related. A previous study showed that *pks*-positive *K. pneumoniae* have low drug resistance (Lan et al., 2019), and our study with more strains and a richer sample source demonstrated similar results. These results indicate that *pks*-positive strains carry abundant virulence genes and fewer antibiotic resistance genes.

To further explore the scenario under which *K. pneumoniae* acquired *pks* sequences, we constructed a phylogenetic tree. Our results showed that the clustering patterns of the CG65 and CG23 lineage strains between the two trees differed, suggesting that the *pks* sequences of some of these lineage strains were acquired through horizontal gene transfer. In addition, the consistency of the CG380 and CG792 lineage strains between the two trees was observed in this study. This may indicate that the most recent common ancestor of these strains acquired the *pks* sequences by horizontal gene transfer, and then passed the *pks* sequences to the offspring by vertical propagation.

Comparative genomic analysis showed that there were differences in mobile genetic elements between *pks*-positive and *pks*-negative *K. pneumoniae*. Our study found that the prevalence of some insertion sequences differed between the *pks*-positive and *pks*-negative strains. Among them, the IS6 family showed a higher prevalence in this study. Previous studies have shown that the IS*6* family, including IS26 and IS257, plays an important role in the spread of multiple antibiotic-resistance genes (Varani et al., 2021). Therefore, we speculate that the low prevalence of IS6 among *pks*-positive strains may be related to their antibiotic resistance. In addition, repB is the most common replicon types in *pks*-positive strains. IS256 and IS21 are common insertion sequence types in *pks*-positive strains. However, the relationship between them and pks island needs further analysis. Notably, we found that the number and total length of prophages in *pks*-positive strains were significantly lower than those in *pks*-negative strains. Considering that phages can carry antibiotic resistance genes and virulence genes, the differences in the number and total length of prophages between *pks*-positive and *pks*-negative strains may be related to the prevalence of resistance and virulence genes among strains. In addition, Silpe et al. showed that colibactin production by *pks*-positive *E. coli* could induce prophages in bacteria (Silpe et al., 2022). These results suggest a potential complex link between *pks* gene islands and prophages.

Analysis of clinical characteristics showed that multiple factors were associated with *pks*-positive *K. pneumoniae* infection. Previous studies have shown a higher proportion of liver abscesses in patients infected with *pks*-positive strains (Shi et al., 2020). Our study found similar results, with a higher detection rate of *pks-*positive strains than of *pks*-negative strains in samples of abscess origin. It has been shown that the *pks* island is widely present in *E. coli* K1, which causes neonatal infections, and colibactin contributes to the colonization of K1 *E. coli* in the gastrointestinal tract (McCarthy et al., 2015). Notably, in this study, more *K. pneumoniae* infections in the neonatal ward were caused by *pks*-negative *K. pneumoniae*. We hypothesize that the possible reason is that the common *K. pneumoniae* may be sufficient to infect neonates due to their low immunity, thus causing the *pks*-positive *K. pneumoniae* to be less dominant in the infection. A study from China showed that infections caused by hypervirulent *K. pneumoniae* are uncommon in children (Li et al., 2021). Given the close relationship between the *pks* island and hypervirulent *K. pneumoniae*, this may also partially explain the low detection rate of *pks*-positive *K. pneumoniae* in patients younger than 1 year of age in this study. In addition, our study showed that patients infected with *pks*-positive *K. pneumoniae* had a higher proportion of diabetes and abscesses. Studies have shown that diabetes and liver abscess are strongly associated with hvKp infections (Zhang et al., 2016; Liu and Guo, 2019; Namikawa et al., 2023). This support that *pks* islands are closely related to hvKp and may play an important role in hvKp infections.

However, this study had certain limitations. We did not calculate the prevalence of *pks* islands in strains from different specimen sources because of the lack of background information from public databases and because the number of strains from different sources varied greatly. We did not collect the clinical data of all patients with *pks*-negative *K. pneumoniae* infection, but only selected some patients as study subjects by stratified random sampling. In addition, retrospective analysis of the data may also introduce bias. Finally, 79.6% of *pks*-positive *K. pneumoniae* isolates in our study were from China, which may make the conclusions of this study less generalizable.

In summary, through a large-scale analysis, we described the genomic characteristics of *pks*-positive *K. pneumoniae* and the clinical characteristics of *pks*-positive *K. pneumoniae* infection. We found for the first time that the number and fragment length of prophages were lower in *pks*-positive strains than in *pks*-negative strains, and the prevalence of the IS*6* family was higher in *pks*-negative strains than in *pks*-positive strains. Plasmid replicon types differed between the *pks*-positive and *pks*-negative strains. However, further studies on the genetic background of *pks* islands in *K. pneumoniae* are needed to gain a deeper understanding of the evolution of *pks* islands in *K. pneumoniae*. In addition, more bacterial species need to be included to fully understand the transfer and spread of *pks* islands among different bacteria.

## Data availability statement

The original contributions presented in the study are included in the article/Supplementary material, further inquiries can be directed to the corresponding author.

## Author contributions

WL and ZW conceived and designed the project. ZW, YL, and PL collected the genomic data and clinical data and performed the statistical analysis. ZW, BT, and AY performed the genome analysis. ZW wrote the manuscript. WL, ZW, ZJ, and QY revised the manuscript. All authors contributed to the article and approved the submitted version.

## Footnotes

^1^ https://db.cngb.org/

^2^ https://github.com/tseemann/mlst

^3^ https://pubmlst.org/

^4^ https://github.com/tseemann/abricate

^5^ https://github.com/tseemann/snippy
