# Supplementary material for: Genomic and clinical characterization of Klebsiella pneumoniae carrying the pks island
Source: Front Microbiol. 2023 Sep 21;14:1189120. doi: 10.3389/fmicb.2023.1189120 (PMC10551629; doi:10.3389/fmicb.2023.1189120)
Supplement: Supplementary file 1 [file Data_Sheet_1.pdf]

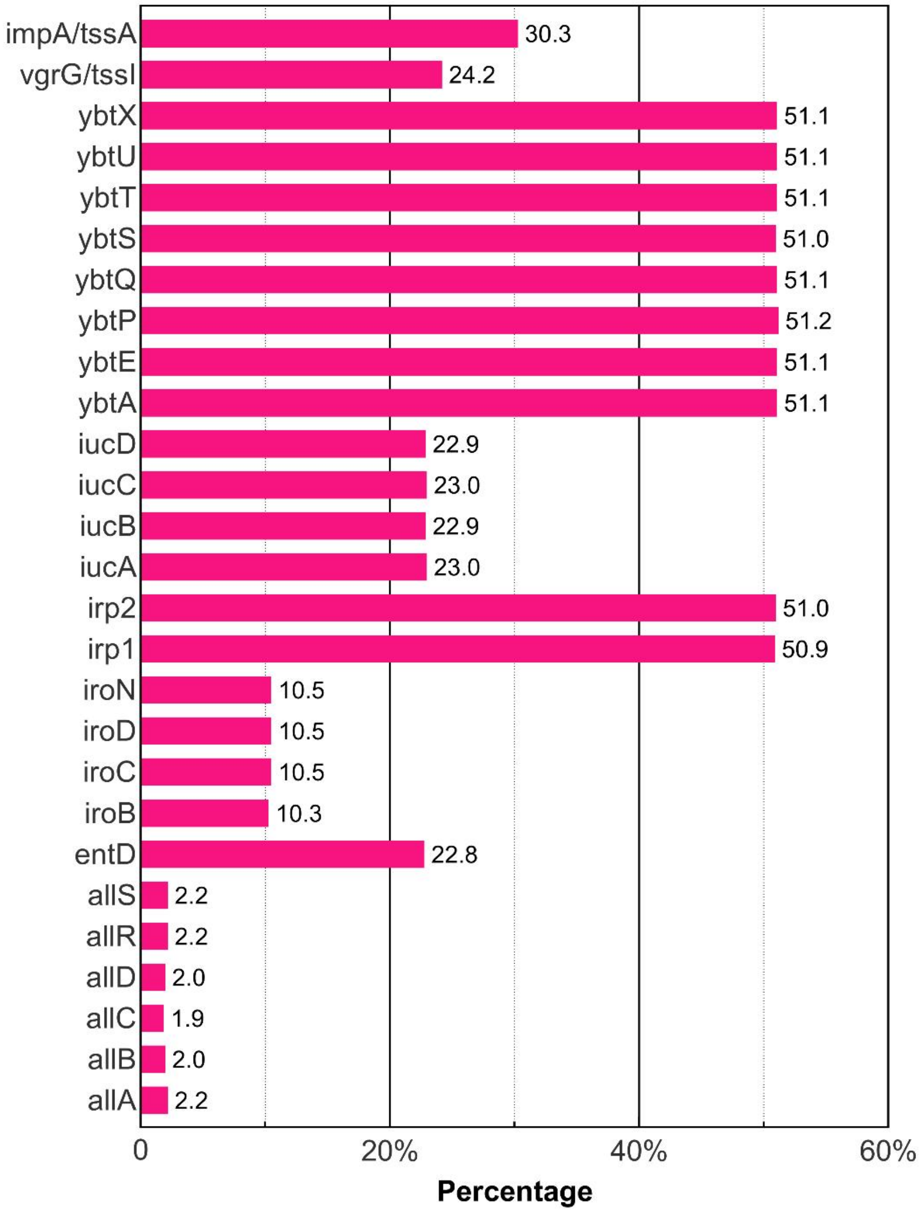

Figure S1 Prevalence of virulence genes in *pks*-negative *Klebsiella pneumoniae*.

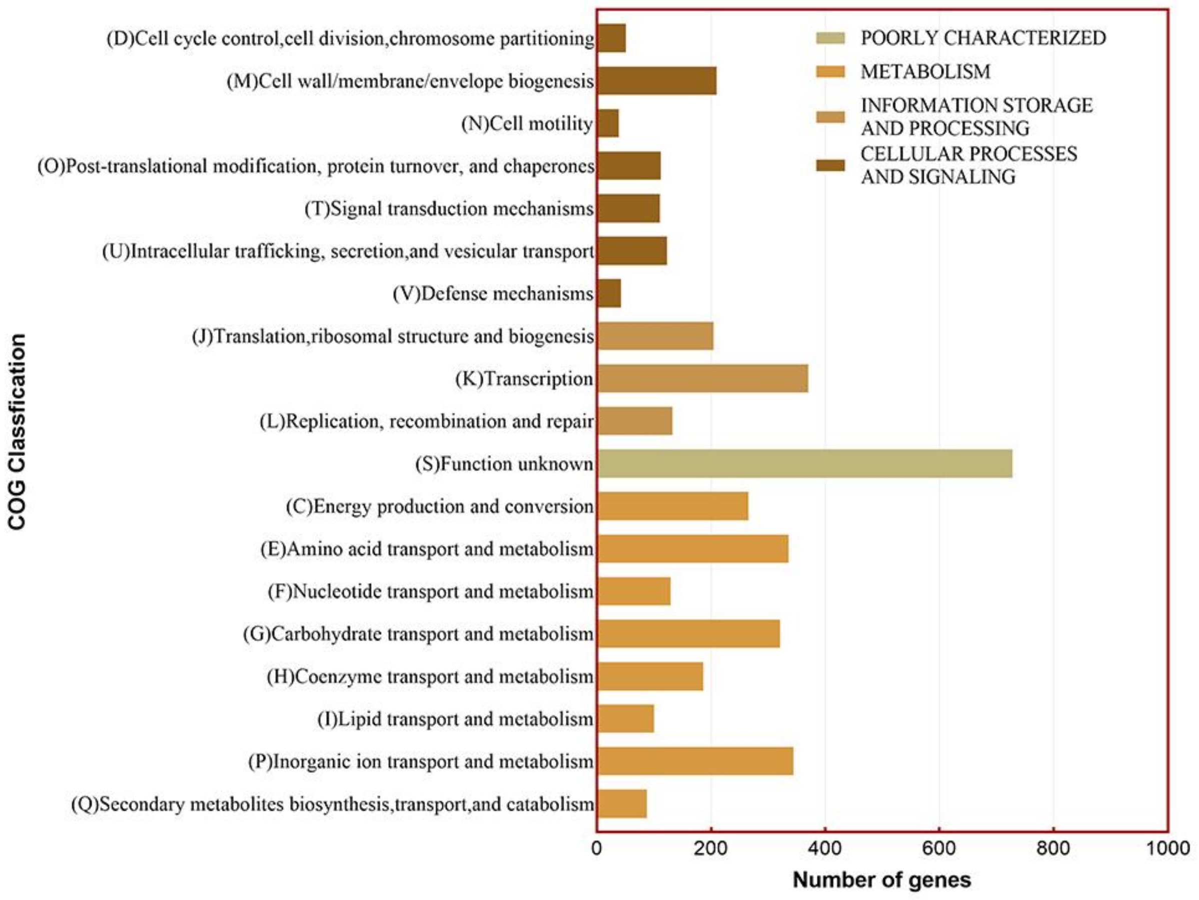

Figure S2 COG classifications for core genes of *pks*-positive strains. The vertical axis represents the different COG classifications and the horizontal axis represents the number of core genes in each COG classification.

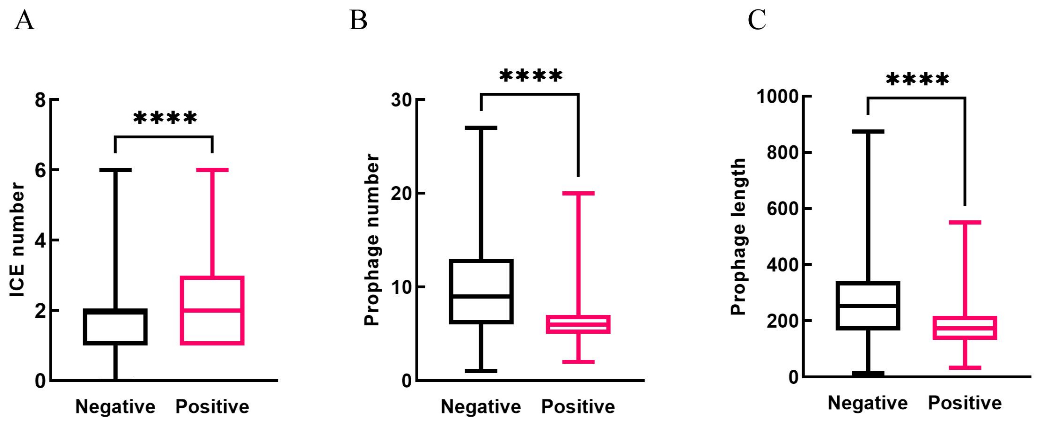

Figure S3 Distribution of predicted ICE and prophage in *pks*-positive and *pks*-negative *K. pneumoniae* genomes (Both complete and incomplete genomes are included). (A) The number of predicted ICE in the genomes of *pks*-positive and *pks*-negative strains. (B) The number of predicted prophage in the genomes of pks-positive and pks-negative strains. (C) The length of predicted prophage in the genomes of pks-positive and pks-negative strains.

Table S3 Distribution of IS family categories in *pks*-positive and *pks*-negative *K. pneumoniae* genomes (complete genomes).

IS Family	*pks*-Negative (n = 1334)	*pks*-Positive (n = 88)	*P* value
IS110	1279 (95.9)	83 (94.3)	0.414
IS5	1255 (94.1)	78 (88.6)	0.063
IS1182	451 (33.8)	29 (32.9)	0.870
IS4	579 (43.4)	13 (14.7)	0.000*
IS1595	6 (0.4)	0 (0.0)	1.000
IS1634	5 (0.4)	0 (0.0)	1.000
IS30	428 (32.1)	6 (6.81)	0.000*
IS21	877 (65.7)	76 (86.3)	0.000*
IS3	1333 (99.9)	88 (100.0)	1.000
ISNCY	1323 (99.2)	86 (97.7)	0.190
IS1202	1315 (98.6)	85 (96.5)	0.151
IS256	257 (19.3)	49 (55.6)	0.000*
IS200/IS605	819 (61.4)	30 (34.0)	0.000*
IS6	1159 (86.9)	49 (55.6)	0.000*
IS630	1332 (99.9)	88 (100.0)	1.000
ISKra4	309 (23.2)	15 (17.0)	0.185
ISAs1	91 (6.8)	3 (3.40)	0.212
IS481	825 (61.8)	41 (46.5)	0.005*
IS1380	857 (64.2)	12 (13.6)	0.000*
ISL3	949 (71.1)	41 (46.5)	0.000*
Tn3	1334 (100.0)	88 (100.0)	NA
IS66	1023 (76.7)	76 (86.3)	0.036*
IS1	1260 (94.5)	82 (93.1)	0.630
IS91	318 (23.8)	16 (18.1)	0.225

*A *P* value <0.05 was considered to be statistically significant. NA: not available

Table S4 Distribution of major plasmid replicon types in *pks*-positive and *pks*-negative *K. pneumoniae* genomes (complete genomes).

Replicon types	pks - negative (n = 1334)	pks - positive (n = 88)	*P*
IncHI1B(pNDM-MAR)	312 (23.4)	47 (53.4)	0.000*
repB	161 (12.1)	52 (59.1)	0.000*
ColRNAI	263 (19.7)	20 (22.7)	0.493
IncFIB(pQil)	122 (9.1)	6 (6.8)	0.460
IncFII(K)	217 (16.3)	19 (21.6)	0.194
IncR	520 (39.0)	6 (6.8)	0.000*
IncX3	99 (7.4)	12 (13.6)	0.035*
IncC	125 (9.4)	3 (3.4)	0.058
IncFIB(pNDM-Mar)	144 (10.8)	0 (0.0)	0.001*
IncL	107 (8.0)	2 (2.3)	0.050
IncFIB(K)	597 (44.8)	21 (23.9)	0.000*
IncFIA(HI1)	110 (8.2)	6 (6.8)	0.636
IncFII(pHN7A8)	213 (16.0)	0 (0.0)	0.000*

*A P value <0.05 was considered to be statistically significant.
